# Role of Multimodal Imaging in Patients With Suspected Infections After the Bentall Procedure

**DOI:** 10.3389/fcvm.2021.745556

**Published:** 2021-12-01

**Authors:** Martina Sollini, Francesco Bartoli, Roberto Boni, Roberta Zanca, Andrea Colli, Maurizio Levantino, Francesco Menichetti, Mauro Ferrari, Raffaella Berchiolli, Elena Lazzeri, Paola A. Erba

**Affiliations:** ^1^Department of Biomedical Sciences, Humanitas University, Pieve Emanuele, Italy; ^2^IRCCS Humanitas Research Hospital, Rozzano, Italy; ^3^Department of Translational Research and New Technology in Medicine and Surgery, Regional Center of Nuclear Medicine, Azienda Ospedaliero Universitaria Pisana, University of Pisa, Pisa, Italy; ^4^Unità Operativa Complessa Medicina Nucleare, ASST Papa Giovanni XXIII, Bergamo, Italy; ^5^Division of Cardiovascular Surgery, Department of Surgical, Medical and Molecular Pathology and Critical Care, Azienda Ospedaliero Universitaria Pisana, University of Pisa, Pisa, Italy; ^6^Infectious Diseases Unit, Department of Clinical and Experimental Medicine, Azienda Ospedaliero Universitaria Pisana, University of Pisa, Pisa, Italy; ^7^Vascular Surgery, Department of Translational Research and Advanced Technology in Medicine, Azienda Ospedaliero Universitaria Pisana, University of Pisa, Pisa, Italy; ^8^Department of Nuclear Medicine and Molecular Imaging, Medical Imaging Centre, University Medical Center Groningen, Groningen, Netherlands

**Keywords:** infection, multimodal imaging, Bentall procedure, SPECT/CT, PET/CT, nuclear medicine

## Abstract

**Purpose:** This study aimed to assess the diagnostic performances of multimodal imaging [i.e., white blood cell single-photon emission computed tomography/CT (^99m^Tc-HMPAO-WBC SPECT/CT) and 18-fluoride-fluorodeoxyglucose positron emission tomography/CT ([^18^F]FDG PET/CT)] in patients with suspected infection after the Bentall procedure, proposing new specific diagnostic criteria for the diagnosis.

**Methods:** Between January 2009 and December 2019, we selected within a cardiovascular infections registry, 76 surgically treated patients (27 women and 49 men, median 66 years, and range 29–83 years). All the patients underwent molecular imaging for a suspected infection after the replacement of the aortic valve and ascending aorta according to the Bentall procedure. We analyzed 98 scans including 49 ^99m^Tc-WBC and 49 [^18^F]FDG PET/CT. A total of 22 patients with very early/early suspected infection (<3 months after surgery) were imaged with both the techniques. Positive imaging was classified according to the anatomical site of increased uptake: to the aortic valve (AV), to both the AV and AV tube graft (AVTG) or to the TG, to surrounding tissue, and/or to extracardiac sites (embolic events or other sites of concomitant infection). Standard clinical workup included in all the patients having echocardiography/CT, blood culture, and the Duke criteria. Pretest probability and positive/negative likelihood ratio were calculated. Sensitivity and specificity of ^99m^Tc labeled hexamethylpropylene amine oxime-WBC SPECT/CT (^99m^Tc-HMPAO-WBC SPECT/CT) and [^18^F]FDG PET/CT imaging were calculated by using microbiology (*n* = 35) or clinical follow-up (*n* = 41) as final diagnosis. ^99m^Tc-HMPAO-WBC scintigraphy and [^18^F]FDG PET/CT findings were compared with 95% CIs by using the McNemar test to those of echocardiography/CT, blood culture, and the Duke criteria.

**Results:** Sensitivity, specificity, and accuracy of ^99m^Tc-HMPAO-WBC were 86, 92, and 88%, respectively, with a slightly higher sensitivity for tube graft infection (TGI) as compared to isolated AV and combined AVTG. Overall, sensitivity, specificity, and accuracy of [^18^F]FDG PET/CT were 97, 73, and 90%, respectively. In 22 patients with suspected very early and early postsurgical infections, the two imaging modalities were concordant in 17 cases [10 true positive (TP) and 7 true negative (TN)]. [^18^F]FDG PET/CT presented a higher sensitivity than ^99m^Tc-HMPAO-WBC scan. ^99m^Tc-HMPAO-WBC scan correctly classified as negative three false-positive (FP) PET/CT findings.

**Conclusion:** Our findings supported the use of ^99m^Tc-HMPAO-WBC SPECT/CT and [^18^F]FDG PET/CT in patients with suspicion infection after the Bentall procedure early in the course of the disease onset to confirm the diagnosis and provide a comprehensive assessment of disease burden through the proposed criteria.

## Introduction

Although cardiovascular infections present a relative low incidence, they are burdened by high morbidity and mortality. The number of subjects with evidence of suspected infections is progressively expanding (1) as results of the increased use of prosthetic material including prosthetic valve (PV), cardiac implantable electrical device (CIED), and vascular prosthesis (VP). Cardiovascular infections occurring after the Bentall procedure—it corrects aorta defects with the positioning of a composite aortic graft (i.e., a vascular tube graft attached with a mechanical or biologic valve, aortic valve tube graft (AVTG) are peculiar (2, 3). Infections including graft infection, fistula, abscesses, and mediastinitis are reported among the possible manifestations associated with the Bentall procedure complications (2, 4–8). Although the ideal treatment of an infection after the Bentall procedure consists in the redo of the Bentall root when the thoracic aortic prosthesis is involved (it occurs in about 2% of cases) (9–11), it is really challenging. Indeed, the replacement of the infected graft is burdened by a high mortality (5, 11), particularly in the case of long-lasting infections or severe comorbidities. Alternative strategies comprise aggressive debridement and irrigation (i.e., graft salvage) and conservative medical treatment (i.e., antibiotics) (5, 12). The lack of evidences about the best treatment option determines a controversial management of these patients. Diagnosis requires an interdisciplinary approach, which combined imaging (echocardiography and CT) with clinical and laboratory data including microbiology. However, diagnosis might be really challenging both in the acute setting due to the presence of confounding factors (i.e., inflammatory postsurgical fluid and air collections in the operative site) (13) and in the case of late infections for the presence of non-specific clinical manifestations. The diagnosis based on the use of the sole morphological imaging has changed during the last years. This (r)evolution has been the consequence of the availability of hybrid imaging. Moreover, functional multimodal imaging has become the standard of care in several clinical conditions including cardiovascular infections (14, 15). Autologous radiolabeled leukocytes white blood cell single-photon emission CT/CT (^99m^Tc-HMPAO-WBC SPECT/CT) and 18-fluoride-fluorodeoxyglucose PET/CT ([^18^F]FDG PET/CT) significantly contribute to infection diagnosis. The first technique is based on the time-dependent accumulation of directly radiolabeled autologous leukocytes in the site(s) of infection. On the other hand, PET/CT is typically performed as a single “shot” acquisition point approximately 1 h after the administration of [^18^F]FDG, which is actively took up by the inflammatory cells activated at the site(s) of infection (1). Currently, the use of functional molecular imaging is recommended in prosthetic valve endocarditis (16, 17) and it is suggested in case of vascular prosthesis infections (14, 18). Very few data, which mainly describe the use of PET/CT, are available on the use of multimodal imaging in patients with infections after the Bentall procedure (19). This study aimed to assess the diagnostic performances between ^99m^Tc-HMPAO-WBC SPECT/CT and [^18^F]FDG PET/CT in patients with suspected infection after the Bentall procedure, proposing new specific diagnostic criteria for the diagnosis.

## Materials and Methods

### Patients

Between January 2009 and December 2019, we selected out of a large series of patients prospectively enrolled in a cardiovascular infections registry, 76 patients (27 women and 49 men, median 66 years, and range 29–83 years). The registry actually included 550 patients who performed molecular imaging for a suspected cardiovascular infection (standard imaging negative of inconclusive), to define the disease burden, in the clinical suspicious of distant sites of infection, and/or to identify the portal of entry of infection. For each patient enrolled in the registry, we collected the following information: (i) demographic data, risk factors, and comorbidities; (ii) type of suspected cardiovascular infection; (iii) date and type of cardiovascular surgery (if any); (iv) concomitant medications; (v) results of clinical examinations, blood tests, blood cultures, standard imaging, and molecular imaging; (vi) management of patient including date and type of treatment (medical or surgical approach); (vii) data about final diagnosis (microbiology and/or clinical examinations); and (viii) data about follow-up, overall survival, date, and cause of death (if applicable). In case of very early/early (<1 and <3 months after surgery, respectively) suspected infection, patients enrolled in the registry are imaged with both the techniques in a maximum of 7 days apart. Conversely, in patients with no very early/early suspected infection, the decision whether to perform [^18^F]FDG PET/CT and radiolabeled WBC is generally taken based on the clinical background, on patient clinical condition/compliance, and the pretest probability of infection. Patients included in the present analysis were selected from the registry if they performed molecular imaging for a suspected infection after the Bentall procedure (mean time from the surgery 41.9 months, range 1 month to 17 years) and had all the registry data available. We analyzed 98 scans including 49 ^99m^Tc-HMPAO-WBC and 49 [^18^F]FDG PET/CT since 22 patients with very early/early (<3 months after surgery) suspected infection were imaged with both the techniques in a maximum of 5 days apart. ^99m^Tc labeled hexamethylpropylene amine oxime-WBC (^99m^Tc-HMPAO-WBC) or [^18^F]FDG PET/CT were performed within 2–8 days from the clinical suspicion of infection. All the patients underwent clinical examination and blood tests including WBC counts, C-reactive protein (CRP), erythrocyte sedimentation rate (ESR), acute phase proteins, electrophoresis, and urinalysis. All the patients underwent three sets of blood cultures (at least one aerobic and one anaerobic) from a peripheral vein as for good clinical practice (20). Other imaging tests included echocardiography [either transthoracic echocardiography (TTE) and transesophageal echocardiography (TEE) alone or in combination], contrast enhanced (ce) cardiac-CT, thorax ce-CT, and abdominal ce-CT. MR of the spine was performed in four selected cases. Specific criteria used for image interpretation are detailed below. Table 1 summarizes the main clinical characteristics and risk factors of patients included in the analysis.

**Table 1:** Summary of the characteristics of the Bentall patients.

**Age (years)**	**Mean ± SD**	**Median**	**Range**	
	62.1 ± 17.6	66	29–83	
Sex	Women	Men		
	27/76 (36%)	49/76 (64%)		
Risk factors	Diabetes	Renal failure	Cutaneous lesions	
	15/76 (19%)	12/76 (16%)	5/76 (7%)	
Blood tests	ESR >n.v.	CRP>n.v.	Leukocytosis	
	39/76(51%)	51/76 (67%)	21/76 (18%)	
Blood culture	Positive		Negative	
	41/76 (54%)		35/76 (46%)	
	*Streptococcus aureus*	13		
	MSSA	7		
	MRSA	6		
	*Staphylococcus* spp.	11		
	*Enterococcus* spp	4		
	*P. aeruginosa*	3		
	Haemophilus	2		
	Candida	2		
	Polimicrobic infections	6		
Duke criteria	Definite	Possible	Rejected	
	11/76 (14%)	35/76 (46%)	30/76 (40%)	
Cardiac-CTA (*n* = 34)	Positive	Negative	Inconclusive	
	7/42 (21%)		16/34 (47%)	11/34 (32%)
	Vegetation			
	Fistula	2/7 (29%)		
	Abscesses	2/7 (29%)		
	Pseudoaneurysms	2/7 (29%)		
	Perforation	–		
	Valve aneurysm	1/7 (13%)		
	Dehiscence of the prosthetic valve	–		
Thorax-CTA	Positive	Negative	Inconclusive	
	27/76 (36%)	32/76 (42%)	17/76 (22%)	
	Peri-graft gas	4/26 (15%)		
	Peri-graft fluid	4/26 (15%)		
	Pseudo-aneurysm formation	10/26 (39%)		
	Aneurysm expansion 8/26 (31%)			
Time from surgery (month)	≤ 1	1–3	4–12	
	7/76 (9%)	15/76 (20%)	11/76 (14%)	
Finaldiagnosis	AVTG infection	No AVTG infection	
	49/76 (64%)			
	Isolate AVTG infections	With metastatic infection/embolism	Firm alternative diagnosis local/systemic infections	No diagnosis

*ESR, erythrocyte sedimentation rate; CRP, C-reactive protein; MSSA, methicillin-sensitive Staphylococcus aureus; MRSA, methicillin-resistant Staphylococcus aureus; Cardiac-CTA, cardiac-CT angiography; Thorax-CTA, thorax-CT angiography; AVTG, aortic valve tube graft*.

### Echocardiography

Transthoracic echocardiography and TEE were performed according to standard procedure as for good clinical practice (21) and reported according to the 2015 European Society of Cardiology (ESC) guidelines (16).

### Contrast-Enhanced Gated Cardiac CT Angiography (CTA) and Thorax CT

Electrocardiogram-gated cardiac CTA was performed after the intravenous (IV) injection of beta-blockers in patients without contraindications (sepsis and unstable patients) and fast heart rate (>65 bpm). The routine protocol for contrast injection consisted of 50–120 ml of isomolar iodinated contrast medium at a flow rate of 4–7 ml/s followed by a 30–50-ml saline chaser. A dedicated software was used for image processing on an independent workstation (Advantage Windows 3.1 or 4.1; Sun Microsystems, Mountain View, California, USA). The presence of vegetations, abscess, pseudoaneurysm, and/or fistula were considered suggestive for IE (22, 23).

Computed tomography scanning of the thorax was performed by multidetector CT (LightSpeed Plus; GE Medical Systems, Oslo, Norway) in all the patients—able to perform a breath hold—with an acceptable renal function (glomerular filtration rate ≥ 60 ml/min/1.73 m^2^). Unenhanced images were followed by the arterial phase acquired after the IV administration of 120 ml iodinated contrast agent at a flow rate of 3–4 ml/s (delay calculated following a bolus test) and after delay of 80–100 s by using a 3-mm collimation and a 1-mm reconstruction spacing with single-row equipment or a 2.5-mm collimation and a 1.25-mm reconstruction spacing. The presence of fistula, pseudoaneurysm, intergraft thrombus, perigraft fluid, perigraft air, perigraft soft-tissue attenuation, and discontinuity of the aneurysmal wrap were considered predictive for the presence of tube graft (TG) infection (24). Other foci of infection (e.g., lung, spleen, spine) were assessed by using the common diagnostic criteria and imaging modalities as appropriate.

### Radiopharmaceuticals and Acquisition Protocols

#### Radiolabeling of Autologous Leukocytes and Image Acquisition Protocol

Autologous WBCs were radiolabeled with ^99m^Tc-HMPAO according to the European Association of Nuclear Medicine (EANM) guidelines (25, 26). Radiolabeling efficiency ranged between 70 and 85%. Viability of radiolabeled leukocytes was routinely checked before the reinfusion by the trypan blue exclusion test. ^99m^Tc-HMPAO-WBC imaging was performed as previously detailed (27, 28). Total-body and spot planar images were acquired 30 min (early), 4–6 h (late), and 20–22 h (delayed) after the reinfusion of ^99m^Tc-HMPAO-WBC (370–555 MBq). Additionally, SPECT/CT images of the chest were obtained by using a dual-head, variable-angle SPECT/CT gamma camera (Hawkeye, GE Healthcare, Oslo, Norway) at 6 h and repeated in negative or doubtful cases at 20–22 h. Both CT attenuation corrected and non-corrected (AC and NAC, respectively) images were analyzed. The Xeleris workstation (Hawkeye, GE Healthcare, Oslo, Norway) was used to fuse the matching pairs of X-ray transmission and radionuclide emission images and to generate the hybrid images of overlying transmission and emission data. Images were displayed and analyzed in axial, coronal, and sagittal planes. Tridimensional maximum intensity projection (MIP) images were available for review.

#### 18-Fluoride-Fluorodeoxyglucose and Image Acquisition Protocol

The administered activity of [^18^F]FDG (Gluscan®, Advanced Accelerator Application, Saint-Genis-Pouilly, France) was of about 3.7 MBq/kg body weight. Patients were fasted for at least 6 h and were prepared—in the 24 h prior to the examination—with a high fat-very low-carbohydrate (HFLC) diet (17). Blood glucose level was performed before the examination and a less strict criterion as compared to oncological patients (<200 and <150 mg/dl, respectively) was accepted as recommended for infection and inflammation (29). PET and CT images were acquired consecutively 60–90 min after the radiopharmaceutical injection of using a PET/CT scanner (Discovery 710; GE Healthcare, Oslo, Norway). CT data were used both for the low-noise attenuation correction of PET emission data and for fusion with attenuation-corrected PET images. PET data were reconstructed iteratively by using ordered-subset expectation maximization software. Images were displayed and analyzed in axial, coronal, and sagittal planes. MIP images were available for study.

### Functional Imaging Interpretation Criteria

Images were independently reviewed by the two experienced nuclear physicians aware of the medical history of patient and of the results of other tests/examinations. Both the ^99m^Tc-HMPAO-WBC and PET/CT images were analyzed and both the presence and the site of area(s) of abnormal radioactivity accumulation suggestive for infection were recorded as previously described (27, 28). Images without site(s) of abnormal ^99m^Tc-HMPAO-WBC or [^18^F]FDG uptake were classified as negative. ^99m^Tc-HMPAO-WBC images were defined as positive when observed at least one area of abnormal time-dependent uptake—from early planar to delayed images (30). PET/CT images were defined as positive when observed increased [^18^F]FDG uptake (i.e., intensity > surrounding tissue that persist at both the AC and NAC images) in a region rather than the once of physiological. PET/CT findings were classify based on the [^18^F]FDG uptake pattern as: (i) positive with either diffuse or focal and (ii) positive with focal pattern. Semiquantitative parameters as described in literature for cardiovascular infections (31) such as the mean and maximum standard uptake values (SUVs), target-to-mediastinum, target-to-lung, target-to-vascular wall, and target-to-liver ratios were calculated.

Uptake suggestive for infection was further classified regardless of the imaging modality (^99m^Tc-HMPAO-WBC or PET/CT) as pertaining to the aortic valve [infective endocarditis (IE)], to both the aortic valve and aortic TG (IE + TG or to the aortic TG), to surrounding tissues (mediastinitis and sternal osteomyelitis), and/or to extracardiac sites (embolic events or other sites of concomitant infection). All the findings consistent with postsurgical changes based on the intensity, location of uptake, and the time length, since the latest surgical procedures were excluded from the analysis.

### Clinical Classification: The Duke Criteria and New 2015 ESC Diagnostic Criteria for the Diagnosis of IE

**Figure 1:**
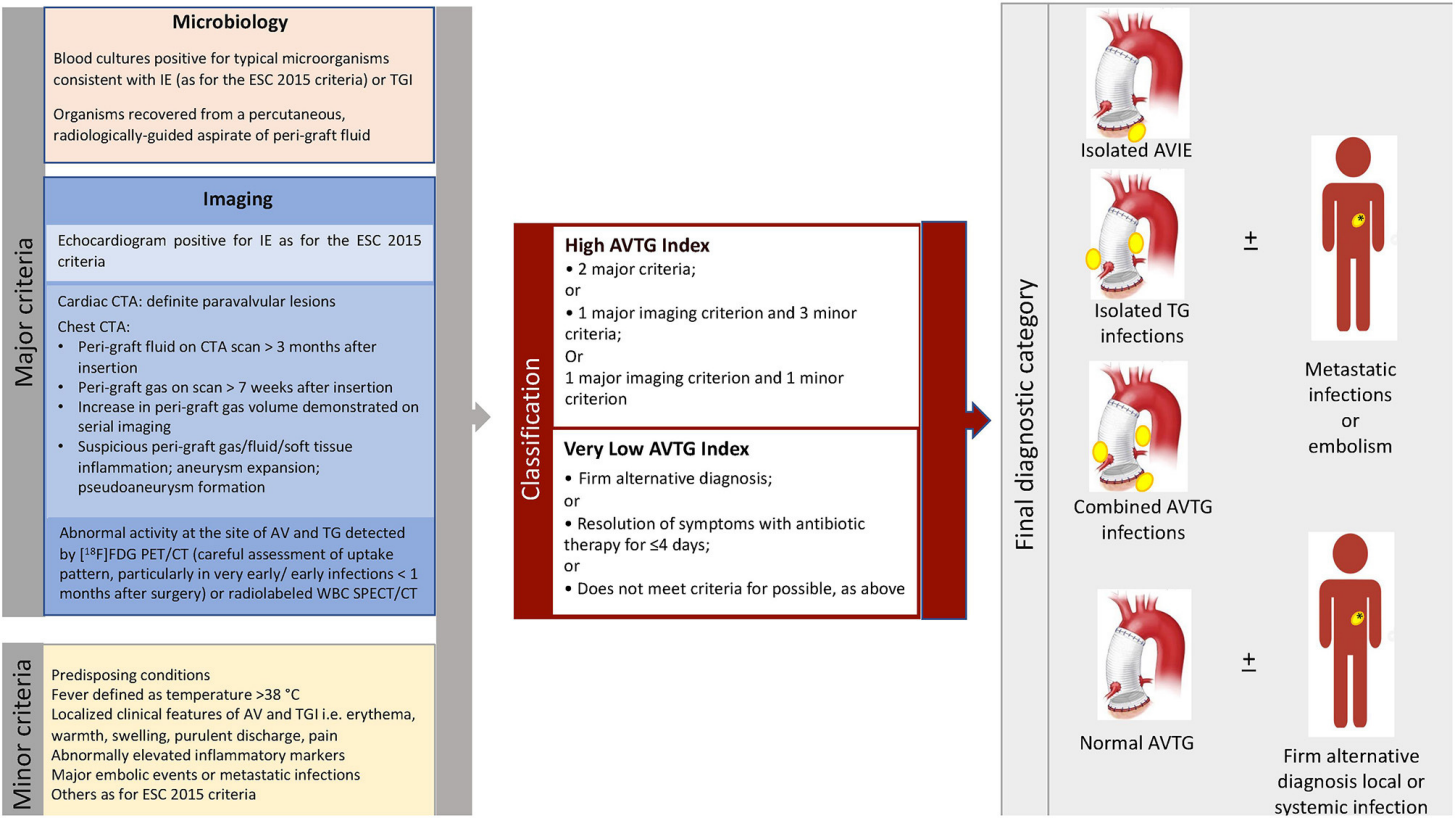
Summary of the major and minor criteria for the diagnosis of aortic valve and aortic tube graft infection proposed (aortic valve tube graft criteria, left panel), interpretation (middle panel), and final classification of patients based on their application (right panel). AVTG, aortic valve tube graft; CTA, CT angiography; [^18^F]FDG, 18-fluoride-fluorodeoxyglucose; IE, infective endocarditis; SPECT, single-photon emission CT; TGI, tube graft infection.

The Duke criteria were used to classify patients (Table 1). Moreover, each patient was classified according to the 2015 ESC criteria, which incorporate the results of the molecular imaging tests. Finally, a new classification including the presence of both aortic valve and aortic tube graft infection [aortic valve tube graft (AVTG) criteria] was developed (Figure 1).

### Final Diagnosis and Follow-Up of Patients

Final diagnosis was obtained by the surgical procedures and microbiological confirmation in 35 patients and by clinical and imaging follow-up in the remaining 41 patients. Surgery was not performed due to major contraindication (*n* = 15), controlled local infection (*n* = 13), fast progression (*n* = 9), and decision of patients (*n* = 4). Management and changes in management of patients based on molecular imaging were recorded. Clinical and outcome data included: (i) type and duration of antimicrobial therapy, (ii) perioperative and long-term mortality, and (iii) infection eradication at 1 year. Recurrence rate was also calculated. The negativity of clinical examination, blood tests (including microbiology), ultrasound (US)/CT scan, and ^99m^Tc-HMPAO-WBC and/or [^18^F]FDG PET/CT imaging were the criteria used to define tube graft infection (TGI) eradication.

All the patients with a negative scintigraphy or PET/CT underwent 3 and 6 month clinical and imaging follow-up with echocardiography and thorax ce-CT.

### Statistical Analysis

Descriptive statistics was used to describe the population. All the values were expressed as median and range. Pretest probability and positive/negative likelihood ratio were calculated. Final microbiological or clinical diagnosis was used to calculate sensitivity and specificity of the Duke classification, ^99m^Tc-HMPAO-WBC SPECT/CT, and [^18^F]FDG PET/CT imaging. Further, ^99m^Tc-HMPAO-WBC scintigraphy and [^18^F]FDG PET/CT findings were compared with 95% CIs by using the McNemar test to those of echocardiography/CT, blood culture, and the Duke criteria. Agreement between the Duke criteria, the 2015 new ESC criteria, and the new AVTG was evaluated by using the exact McNemar test. Parametric data were compared with the Pearson's χ^2^ test. All the PET/CT semiquantitative parameters calculated were compared by using the Mann–Whitney *U* test in the following groups: positive and negative scans, patients with IE/patients without IE, patients with TG/patients without TG, and patients with combined IE/TGI.

## Results

**Figure 2:**
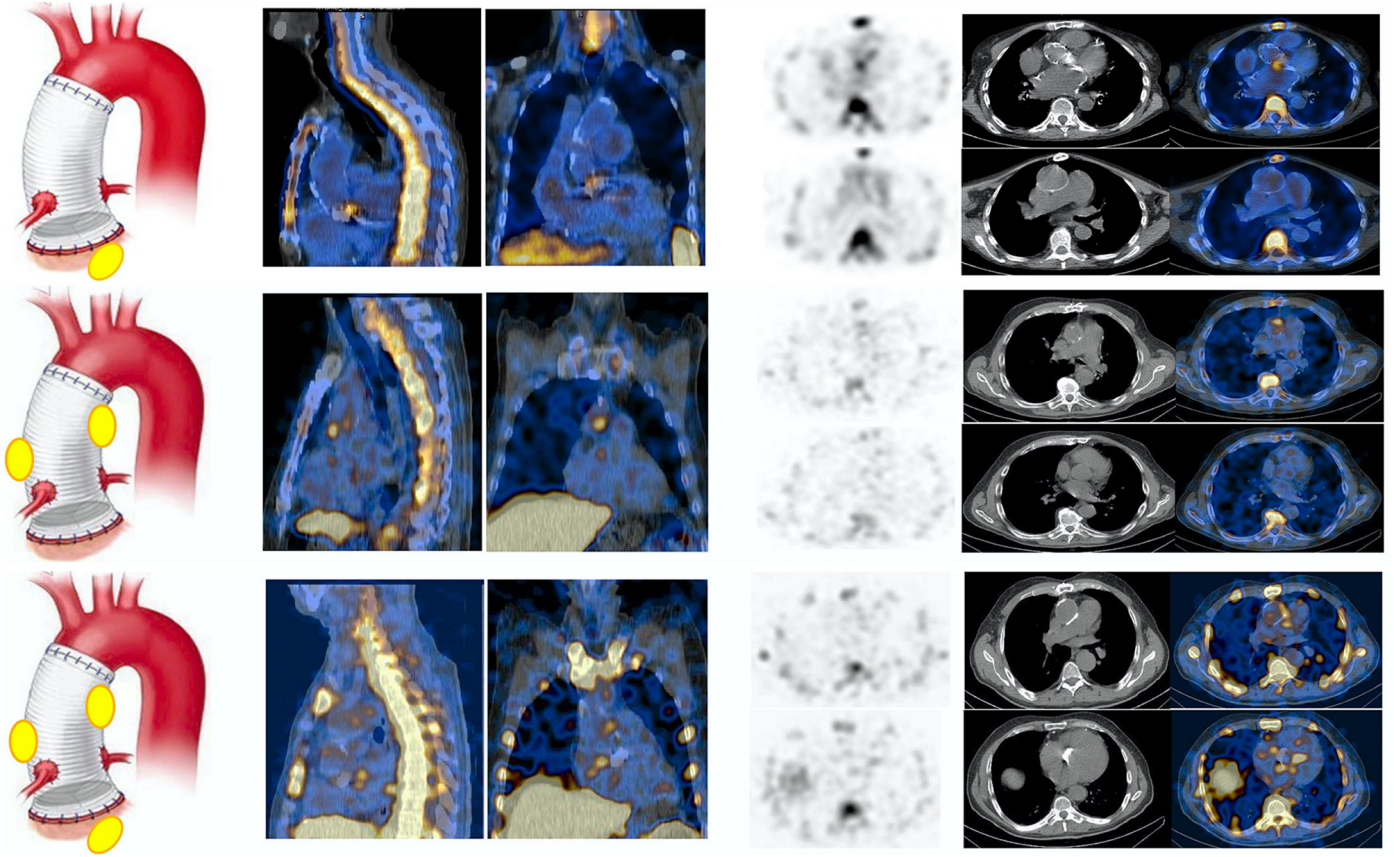
Example of white blood cell (^99m^Tc-HMPAO-WBC) scan in patients with final diagnosis infection involving the AV (upper panel), the TG (middle panel), and at both the AV and TG (AVTG, lower panel) as shown by the SPECT/CT images of the thorax (from left to right, superimposed sagittal and coronal and transaxial emission, CT and superimposed SPECT/CT images at both the TG and the AV levels) showing area of focal radiopharmaceutical uptake involving either the aortic prosthetic valve, the tube graft, or both. The left panel of the figure shows a schematic representation of the final diagnostic category.

According to final diagnosis, a total of 49/76 (64%) patients were finally diagnosed with AVTG infection (Table 1). ^99m^Tc-HMPAO-WBC scintigraphy was negative for AVTG infection in 17/49 examinations. In 9 out of 17 scans, alternative sites of infections were found (see below). One patient was FP. Table 2 details the diagnostic performances of ^99m^Tc-HMPAO-WBC scintigraphy. At least one abnormal area of pathological radiolabeled WBC uptake at AV and/or TG was detected in 32/49 scans including 5 IE, 13 IE extending to the TG, and 14 involving the TG (Figure 2). Overall, sensitivity, specificity, negative predictive value (NPV), positive predictive value (PPV), and accuracy of ^99m^Tc-HMPAO-WBC were 86, 92, 71, 97, and 88%, respectively (Table 2), with a slightly higher sensitivity for TGI as compared to isolated AV and combined AVTG.

**Table 2:** Diagnostic performance of ^99m^Tc-HMPAO-WBC scintigraphy (*n* = 49 scans).

	**AVTG infection**				**Whole body assessment**	
	**Isolated AVIE**	**TGI**	**AVIE+ TGI**	**Total**	Metastatic infections or embolism associated to other findings^*^	**Metastatic infections or embolism (unique site)**
TP	5	14	13	31	18	8
TN	42	35	34	12		
FP	0	0	0	1		
FN	2	1	2	5	4	1
Sensitivity	0.71	0.93	1.00	0.86	
Specificity	1.00	1.00	0.96	0.92	
Accuracy	0.96	0.98	1.00	0.88	
PPV	1.00	1.00	0.94	0.97	
NPV	0.95	0.97	–	0.71	
+LR	–	–	0.13	11.19	
–LR	0.29	0.07		0.15	

*^99m^Tc-HMPAO-WBC, ^99m^Tc labeled hexamethylpropylene amine oxime-white blood cell; AVTG, aortic valve tube graft; AVIE, aortic valve infective endocarditis; TGI, tube graft infection; TP, true positive; TN, true negative; FP, false positive; FN, false negative; PPV, positive predictive value; NPV, negative predictive value; +LR, positive likelihood ratio; –LR, negative likelihood ratio*.

### Visual and Semiquantitative Imaging Assessment

**Figure 3:**
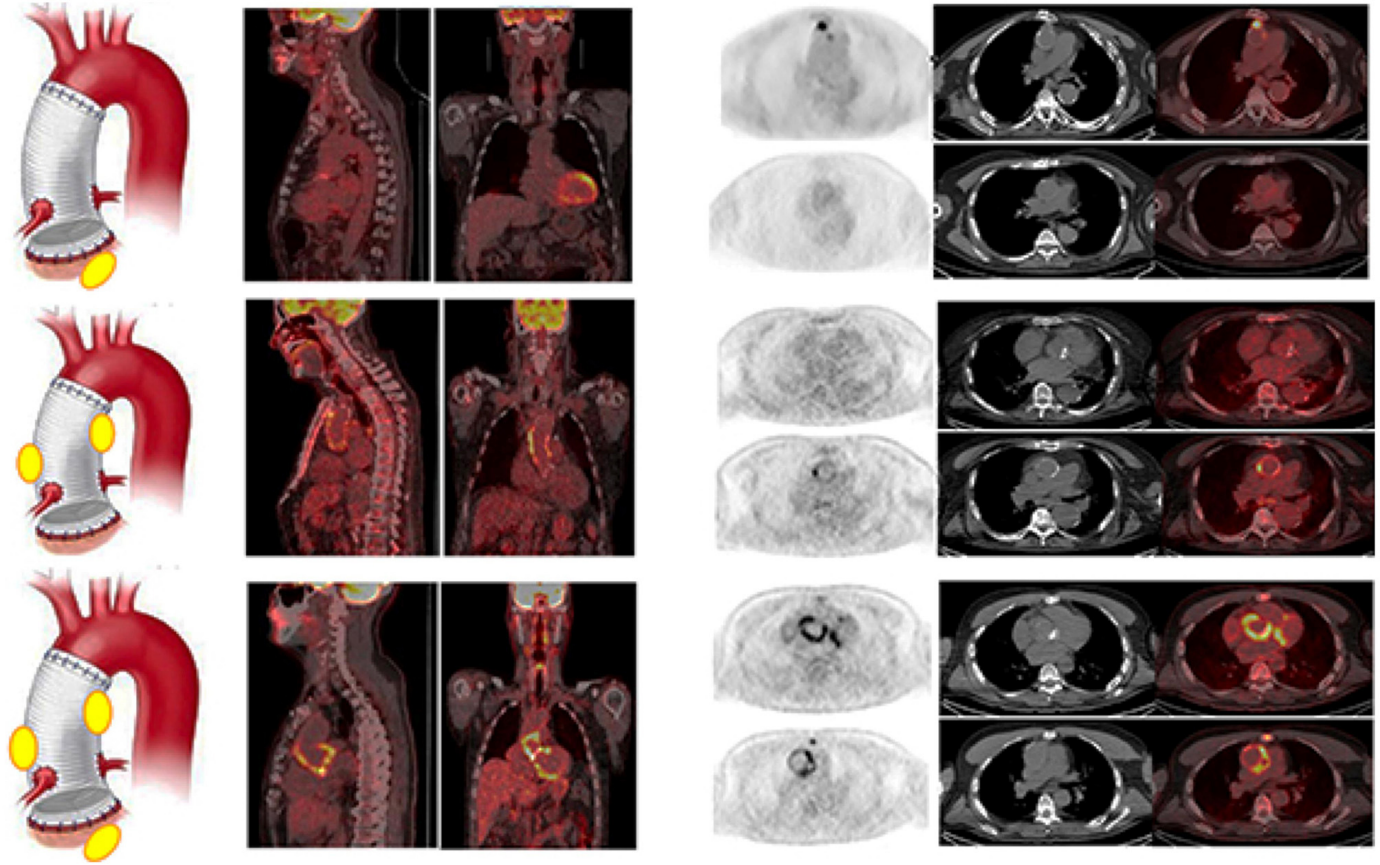
Examples of [^18^F]FDG PET/CT in patients with final diagnosis infection involving the AV (upper panel), the TG (middle panel), and at both the AV and TG (AVTG, lower panel) as shown by the PET/CT images of the thorax (from left to right, superimposed sagittal and coronal and transaxial emission, CT and superimposed images at both the TG and the AI levels) showing area of focal radiopharmaceutical uptake involving limited to the aortic prosthetic valve, to the tube graft, or involving both the component of the composite prosthesis. The left panel of the figure shows a schematic representation of the final diagnostic category.

All the patients were adequately prepared with a 24-h HFLC diet. We obtained the complete suppression of [^18^F]FDG myocardial uptake in all the scans. [^18^F]FDG PET/CT was rated as positive in 37/49 examinations (Table 3). Four cases resulted false positive (FP). Three out of the four FP [^18^F]FDG PET/CT findings were found in the group of patients with very early infection (<1 month from surgery, see below). Infection limited to the AV and the TG was detected in 6 and 16 cases, respectively (Figure 3). In the remaining 11 cases, PET/CT diagnosed an infection involving both the AV and the TG (Figure 3). Concomitant extravascular sites of infections were found in 22 cases. A negative [^18^F]FDG PET/CT for AVTG infection was found in 12 examinations (see below). The overall sensitivity of [^18^F]FDG PET/CT was 97% with a specificity of 73% and an accuracy of 90% (NPV = 92% and PPV = 89%). Excluding the very early subgroup (<1 month from surgery), [^18^F]FDG PET/CT accuracy scaled up to 95%.

**Table 3:** Diagnostic performance of [^18^F]FDG PET/CT (49 scans).

	**AVTG infection**								**Whole body assessment**	
	**Isolated AVIE**		**TGI**		**AVIE+TGI**		**Total**		**Metastatic infections or embolism associated to other findings**	**Metastatic infections or embolism (unique site)**
	**PET criteria alone**	**PET/CT criteria combined**	**PET criteria alone**	**PET/CT criteria combined**	**PET criteria alone**	**PET/CT criteria combined**	**PET criteria alone**	**PET/CT criteria combined**		
TP	6	6	11	16	8	11	25	33		
TN	41	42	30	31	35	35	8	11	22	12
FP	2	1	7	1	5	2	14	4		
FN	0	0	1	1	1	1	2	1	4	0
Sensitivity	1.00	1.00	0.91	0.94	0.89	0.92	0.93	0.97		
Specificity	0.95	0.98	0.81	0.97	0.88	0.95	0.36	0.73		
Accuracy	0.96	0.98	0.84	0.96	0.88	0.94	0.67	0.90		
PPV	0.75	0.86	0.61	0.94	0.62	0.85	0.64	0.89		
NPV	1.00	1.00	0.97	0.97	0.97	0.97	0.80	0.92		
+LR	21.5	43	4.85	30.12	7.11	16.96	1.46	3.64		
–LR	0.0	0.0	0.10	0.06	0.13	0.09	0.20	0.04		

*[^18^F]FDG, 18-fluoride-fluorodeoxyglucose; AVTG, aortic valve tube graft; AVIE, aortic valve infective endocarditis; TGI, tube graft infection; TP, true positive; TN, true negative; FP, false positive; FN, false negative; PPV, positive predictive value; NPV, negative predictive value; +LR, positive likelihood ratio; –LR, negative likelihood ratio*.

**Figure 4:**
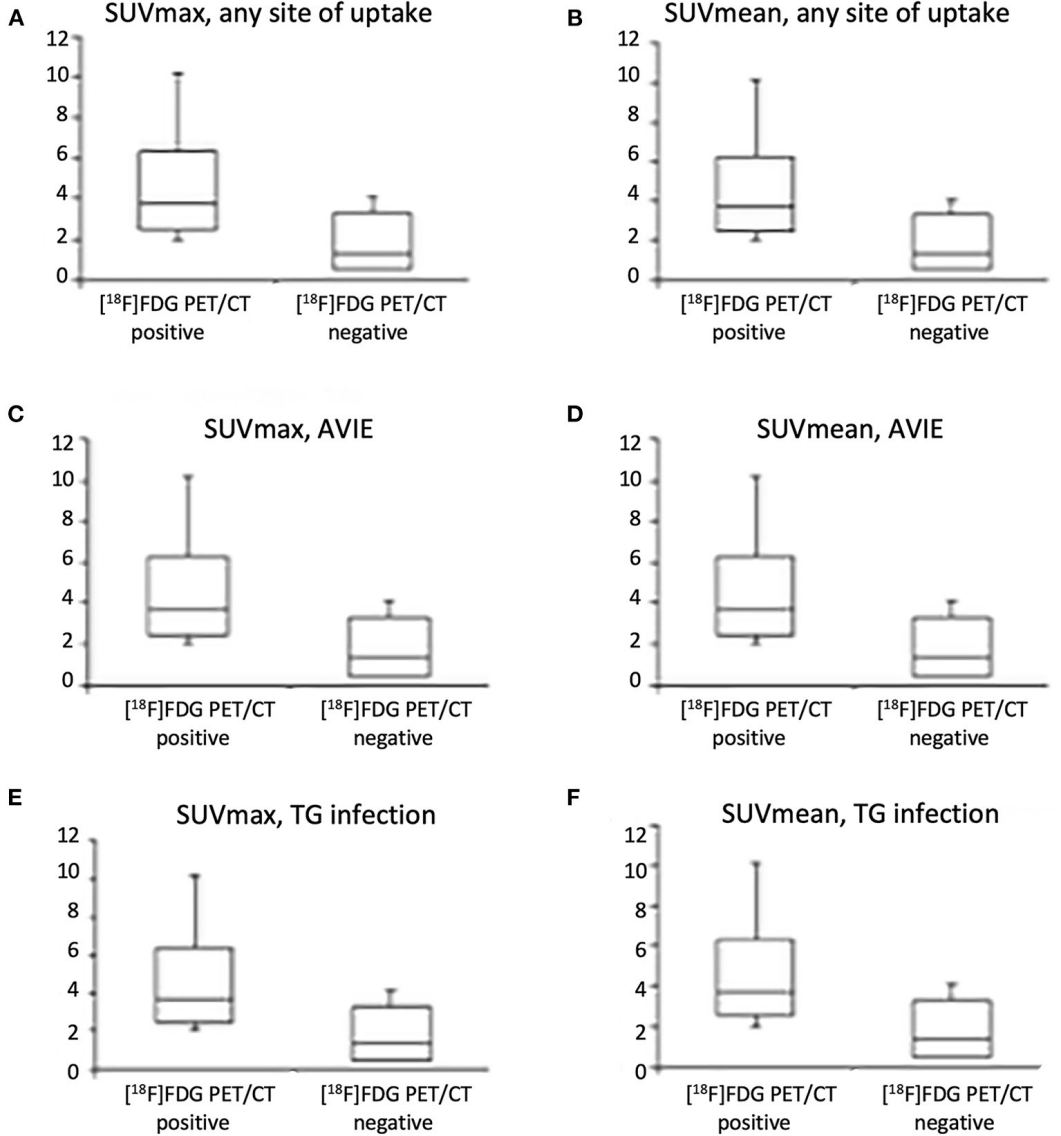
Box plot shows maximum standardized uptake value (SUVmax) and SUVmean in our cohort when considering any site of uptake **(A,B)**, uptake at valve site consistent with IE **(C,D)**, and uptake in infected and non-infected vascular prosthesis infection (VPI) **(E,F)**.

Maximum standard uptake values and SUVmean were higher in patients with a positive scan or with IE/TG than patients with a negative scan or without infection, respectively (Table 4, Figures 4A–F, *p* <0.001). The target-to-lung ratio achieved the highest accuracy in differentiating patients with and without infections. However, none of the semiquantitative parameters alone perform better than the visual assessment.

**Table 4:** Semiquantitative parameters obtained by [^18^F]FDG PET/CT.

	**SUV_**max**_**	**Ratio SUV_**max**_/liver**	**Ratio SUV_**max**_/lung**	**Ratio SUV_**max**_/mediastinum**	**Ratio SUV_**max**_/muscle**	**Ratio SUV_**max**_/caval vein**
Mean	5.6	1.5	6.9	2.3	4.0	5.6
Medium	4.6	1.3	5.0	1.4	3.3	4.6
SD	2.7	0.7	5.0	1.6	1.9	2.7
Range	2.5	0.9	1.9	1.0	2.3	2.5

*[^18^F]FDG, 18-fluoride-fluorodeoxyglucose; SUV_max_, maximum standardized uptake value*.

Table 5 shows the results of ^99m^Tc-HMPAO WBC and [^18^F]FDG PET/CT based on the Duke criteria in all the patients according to the results and further subcategorized for the site of radiopharmaceuticals uptake. The group of patients more often positive at ^99m^Tc-HMPAO-WBC and PET/CT in the possible (*n* = 26) and rejected (*n* = 26) Duke category presented infections not limited to the valvular component of the prosthesis. However, also in patients with the define Duke criteria in 5/11 cases, the infection extended over the valve to the TG. Taking all together, these findings underlie the need of a more comprehensive assessment of these patients not to underdiagnosed infection burden.

**Table 5:** Results of the Duke criteria in ^99m^Tc-HMPAO-WBC and [^18^F]FDG PET/CT based on the site of radiopharmaceutical uptake.

		**AVTG infection**										**Whole body assessment**
		**AVIE**		**TGI**				**AVIE** **+** **TGI**				**Metastatic infections or embolism alone**
		**Isolated**	**Metastatic infections or embolism**	**Isolated**	**Sternal OM**	**Mediastinitis**	**Metastatic infections or embolism**	**Isolated**	**Sternal OM**	**Mediastinitis**	**Metastatic infections or embolism**	
Duke definite	WBC positive	3	3	–	–	–	–	2		1	2	–
	[^18^F]FDG PET/CT positive	3	3					3			1	2
Duke possible	WBC positive	2	1	4	1			9	1	3	3	4^*^
	[^18^F]FDG PET/CT positive	3	2	5		2	1	8^*^		3	4	4
Duke reject	WBC positive	–	–	10^*^	2	3	2	2		1		5
	[^18^F]FDG PET/CT positive	1^*^	1	12^*^	3	1	1	2^*^				5

* *including 1 FP*.

*^99m^Tc-HMPAO-WBC, ^99m^Tc labeled hexamethylpropylene amine oxime-white blood cell; [^18^F]FDG, 18-fluoride-fluorodeoxyglucose; AVTG, aortic valve tube graft; AVIE, aortic valve infective endocarditis; TGI, tube graft infection; OM, osteomyelitis; FP, false positive*.

### Extracardiac/Extravascular Sites

Extracardiac and extravascular sites of infection were overall found in 24/76 patients (34%), in 13 cases as multiple sites (per patient analysis), identifying a subset of more diffuse and complex infections. As for a per site analysis, ^99m^Tc-HMPAO-WBC depicted 8 and 18 distant infection foci either alone or associated with infection of the valve/vascular component of the prosthesis, respectively (Tables 2, 5). Surgical wound infection, sternal osteomyelitis, mediastinitis, and lung infections were most often found. False-negative (FN) results were found in case of extracardiac infections in case of small central nervous system (CNS), lung embolism, and standard deviation (SD) extended to the paravertebral region (*n* = 4). [^18^F]FDG PET/CT identified extracardiac and extravascular findings either alone in 12 sites or associated with valve/vascular uptake in 26 sites, being the most frequent findings in surgical wound infection, mediastinitis, lung infection, osteomyelitis, and mycotic aneurysm (Tables 3, 5). The most common FP findings (*n* = 4) were due to the presence of superficial soft-tissue granuloma and sternal osteomyelitis.

### Patients With Very Early Infections: Comparison of ^99m^Tc-HMPAO-WBC and [^18^F]FDG PET/CT

**Figure 5:**
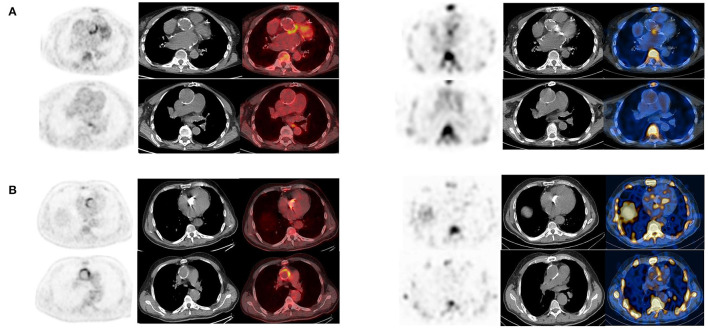
**(A,B)** Examples of concordant findings between [^18^F]FDG PET/CT (left panel, from left to right transaxial emission, CT and superimposed images) and WBC scan (right panel, from left to right transaxial emission, CT and superimposed images) in two patients with very early infection showing uptake of the corresponding radiopharmaceutical at both the imaging modalities localized at the TG **(A)** and at both the AV and the TG **(B)**.

**Figure 6:**
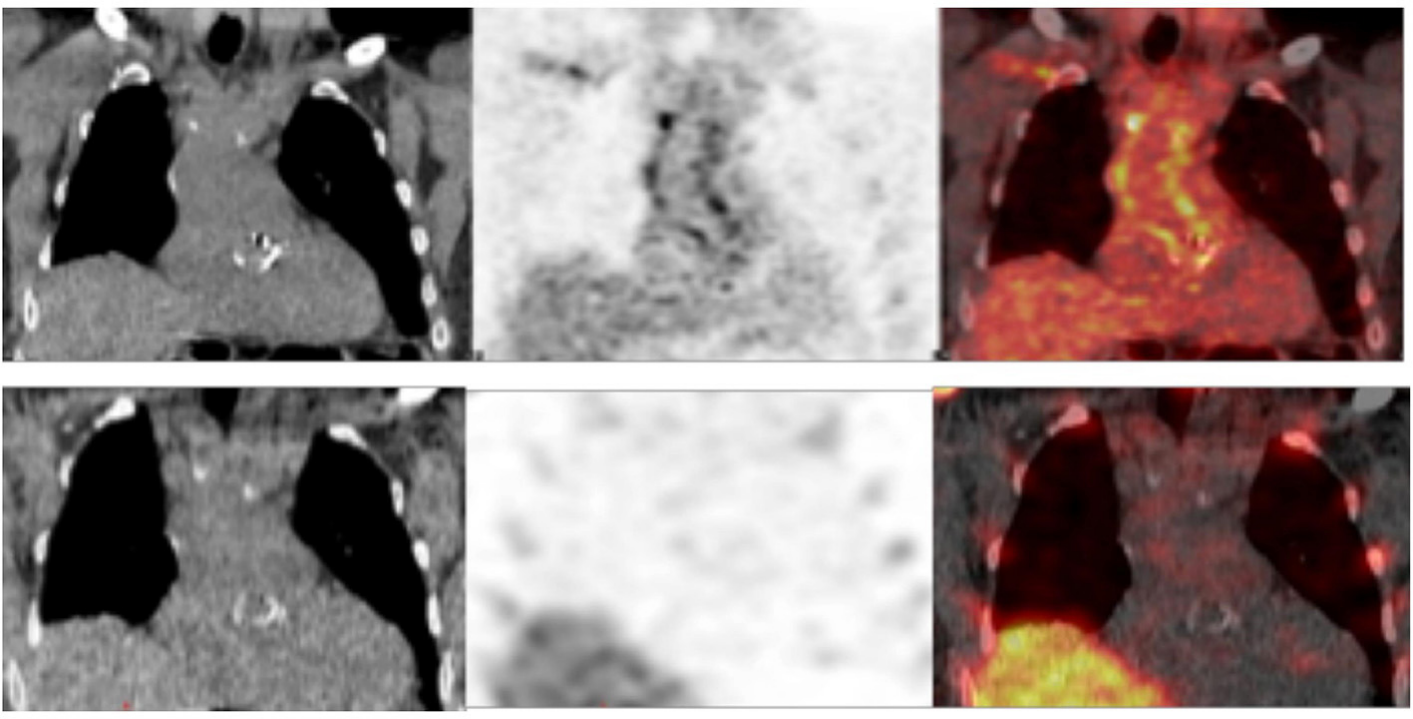
Examples of discordant findings between [^18^F]FDG PET/CT (upper panel, coronal view from left to right CT, emission and superimposed PET/CT) and ^99m^Tc labeled hexamethylpropylene amine oxime-WBC (^99m^Tc-HMPAO-WBC) (lower panel, coronal view from left to right CT, emission and superimposed SPET/CT) in a patient imaged 1 month after the Bentall procedure. PET/CT showed increased [^18^F]FDG uptake at both the aortic valve and the vascular graft (diffuse pattern with areas of focal accumulation), while WBC SPECT/CT results negative. A 2-year follow-up excluded infection.

In 22 patients with suspected very early and early postsurgical infections who underwent both the ^99m^Tc-HMPAO-WBC and [^18^F]FDG PET/CT consecutively, results of the two imaging modalities were concordant in 17 cases [10 true positive (TP) and 7 true negative (TN), Table 6, Figures 5A,B]. [^18^F]FDG PET/CT presented a higher sensitivity than WBC scan (92 and 83%, respectively). WBC scan correctly classified as negative to the three FP PET/CT findings consisting of a focal over a diffuse area of focal [^18^F]FDG uptake along the TG (Figure 6). All these patients presented no signs of infections during the follow-up, thus resulting in a higher specificity and accuracy of WBC. WBC and PET/CT were more beneficial in the possible (*n* = 5) and rejected (*n* = 6), while in the definite Duke category 2 patients presented more extended infection as compared to the initial diagnosis (Table 7).

**Table 6:** Diagnostic performance of ^99m^Tc-HMPAO-WBC SPECT/CT and [^18^F]FDG PET/CT in 22 patients with early/very early infections.

	**^**99m**^Tc-HMPAO-WBC SPECT/CT positive**	**[^**18**^F]FDG PET/CT positive**
TP	10	11
TN	10	7
FP	0	3
FN	2	1
*Sensitivity*	0.83	0.92
*Specificity*	1.00	0.70
*Accuracy*	0.91	0.82
PPV	1.00	0.79
NPV	0.83	0.88
+LR	–	3.06
–LR	0.17	0.12

*^99m^Tc-HMPAO-WBC, ^99m^Tc labeled hexamethylpropylene amine oxime-white blood cell; SPECT, single-photon emission CT; [^18^F]FDG, 18-fluoride-fluorodeoxyglucose; AVTG, aortic valve tube graft; AVIE, aortic valve infective endocarditis; TGI, tube graft infection; TP, true positive; TN, true negative; FP, false positive; FN, false negative; PPV, positive predictive value; NPV, negative predictive value; +LR, positive likelihood ratio; -LR, negative likelihood ratio*.

**Table 7:** Comparative assessment of ^99m^Tc-HMPAO-WBC SPECT/CT and [^18^F]FDG PET/CT in 22 patients with early/very early infections as for site of uptake identified; ^*^including 1 FP in each.

		**AVTG infection**								**Whole body assessment**
		**AVIE**		**TGI**				**AVIE** **+** **TGI**				**Metastatic infections or embolism alone**
		**Isolated**	**Metastatic infections or embolism**	**Isolated**	**Sternal OM**	**Mediastinitis**	**Metastatic infections or embolism**	**Isolated**	**Sternal OM**	**Mediastinitis**	**Metastatic infections or embolism**	
Duke definite	WBC positive	1	3	–	–	–	–	1		1	2	–
	[^18^F]FDG PET/CT positive	1	3					3*			1	–
Duke possible	WBC positive	1	1	2	1			4	1	3	3	3
	[^18^F]FDG PET/CT positive	1	2	2		2	1	3*		2	2	4
Duke reject	WBC positive	–	–	3	2	2	2	1			1	–
	[^18^F]FDG PET/CT positive	–	–	4*	1	1	2	1*			1	3

*^99m^Tc-HMPAO-WBC, ^99m^Tc labeled hexamethylpropylene amine oxime-white blood cell; SPECT, single-photon emission CT; [^18^F]FDG, 18-fluoride-fluorodeoxyglucose; AVTG, aortic valve tube graft; AVIE, aortic valve infective endocarditis; TGI, tube graft infection; OM, osteomyelitis; FP, false positive*.

### Change in the Diagnostic Criteria Category

Overall, sensitivity, specificity, NPV, PPV, accuracy, positive likelihood ratio (+LR), and negative LR (–LR) of the Duke criteria were 38, 58, 33, 63, 45%, 0.90, and 1.07, respectively (Supplementary Table 1 and Table 8). The incorporation of the results of ^99m^Tc-HMPAO-WBC and [^18^F]FDG PET/CT into the Duke criteria, according the recommendation of the 2015 new ESC criteria, determined a decrease of patients previously classified as possible. Those cases were reclassified in the definite (11/35) and the rejected (8/23) categories (Table 8). The 2015 new ESC criteria sensitivity increased to 53%, specificity increased to 76%, NPV increased to 43%, PPV increased to 81%, accuracy increased to 60%, +LR increased to 2.16, and -LR increased to 0.63. The proposed AVTG criteria resulted in the highest diagnostic performance with sensitivity 88%, specificity 81%, NPV 79%, PPV 90%, accuracy 85%, +LR 4.74, and –LR 0.15.

**Table 8:** Comparative assessment of the Duke criteria, the ESC criteria, and the New Bentall 2020 criteria in 76 patients.

	Duke^*^	ESC 2015^*^	Proposed AVTG criteria^*^
TP	19	27	43
TN	15	19	22
FP	11	6	5
FN	31	24	6
Sensitivity	0.38	0.53	0.88
Specificity	0.58	0.76	0.81
Accuracy	0.45	0.60	0.85
PPV	0.63	0.81	0.90
NPV	0.33	0.43	0.79
+LR	0.90	2.16	4.74
-LR	1.07	0.63	0.15

* *Definite and possible of the Duke and the ESC criteria are considered together as positive test*.

*ESC, European Society of Cardiology; AVTG, aortic valve tube graft; TP, true positive; TN, true negative; FP, false positive; FN, false negative; PPV, positive predictive value; NPV, negative predictive value; +LR, positive likelihood ratio; -LR, negative likelihood ratio*.

### Treatment and Follow-Up of Patients

Overall, 35 patients underwent surgery for prosthesis substitution consisting of open surgical repair with a complete resection of the infected graft, thorough debridement of surrounding tissue, and *in-situ* graft replacement. Median operation time was about 310 min (range 265–412 min), median postoperative intensive care unit (ICU) stay was 5 days (range 2–11 days), and median postoperative hospital stay was 21 days (15–23 days). The in-hospital mortality rate was 34% (12/35), being multiorgan failure, mediastinitis, pulmonary causes, bleeding, and wound infections were the main causes of death. Reinfection rate was 4/23 (17%). Midterm survival at 1 year was 82%: two patients died after reoperation for a recurrent infection during follow-up and the other two patients for cause unrelated to the previous infection. In 15/49 cases, including four very early/early infections, surgery was contraindicated and chronic antimicrobial treatment was initiated. In this group, midterm survival at 1 year was 47%.

Patients presenting functional imaging [WBC scan or [^18^F]FDG PET/CT] negative for AVTG were treated with prolonged antimicrobial therapy based on the isolated microorganisms and with specific surgical approach when appropriate (i.e., drainage of the soft-tissue infection, pleural effusion drainage). In four cases due to a mechanical complication, endovascular aneurysm repair (EVAR) treatment was also performed. Among the patients with negative WBC scan/[^18^F]FDG PET/CT, only one developed a mediastinitis (4 months after the first imaging), which was surgically treated. In all the other cases, no signs of infections were further demonstrated during the follow-up.

### ^99m^Tc Labeled Hexamethylpropylene Amine Oxime-WBC and [^18^F]FDG PET/CT Change in Treatment Management

^99m^Tc labeled hexamethylpropylene amine oxime-WBC and [^18^F]FDG PET/CT findings resulted in changes of management of patients in 29/76 (32%) of the cases. Specifically, in 7/29 cases, treatment was modified from best medical treatment to surgery. In 7/29 cases, surgery was contraindicated and chronic antimicrobial treatment was initiated, first as parenteral infusion followed by prolonged oral antimicrobial therapy. All these patients were followed up by CTA and [^18^F]FDG PET/CT imaging to monitor disease evolution. This approach resulted in infection control in three cases, while the other four patients died. In 11 patients presented stable clinical condition and concomitant distant sites of infections (mediastinitis, *n* = 4; lung infections, *n* = 2; mycotic aneurysm, *n* = 1; and multiple infection foci, *n* = 4), surgery was delayed for at least 2 weeks and in the time interval-specific IV antimicrobial treatment was administered and continued after surgery as for specific indication. Surgical management of infected AVTG and concomitant surgical remediation of wound infections, sternal osteomyelitis, and mediastinitis were performed in the other cases.

### Diagnostic Algorithm for Patients With Suspected Infection of a Composite Aortic Valve and Ascending Aorta Prosthesis

**Figure 7:**
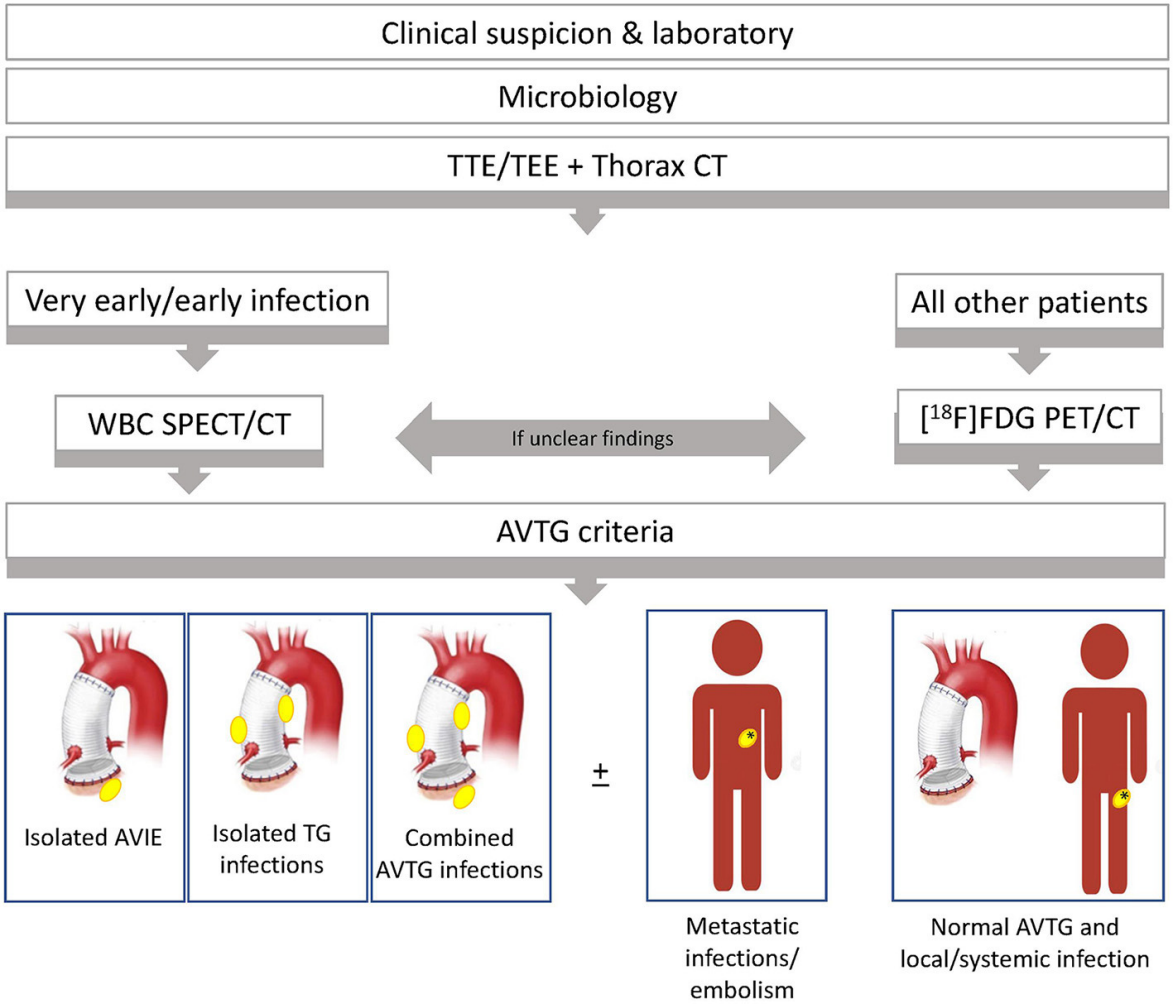
Diagnostic flowchart, currently used in our institutions, developed the result of the study presented here. After a clinical and laboratory assessment for the pretest risk definition, echocardiography [either transthoracic echocardiography (TTE) and transesophageal echocardiography (TEE) followed by cardiac CT angiography (CTA), if needed] and CTA of the thorax are performed. According to our data, functional imaging is recommended whenever equivocal TEE/TTE and cardiac/thoracic CTA are present in high-risk patients to confirm the clinical suspicion of infection. Moreover, the use of functional imaging is supported to properly define the disease burden and prevent understaging, regardless of the positivity/negativity of TEE/TTE and/or CTA. This approach seems of value in complicated patients bearing high risk for surgical procedures. The decision of whether to perform [^18^F]FDG PET/CT and radiolabeled WBC is generally taken based on the clinical background, on clinical condition/compliance of patient, and the pretest probability of infection. As a general rule, radiolabeled WBC is preferred in case of very early and early infections. [^18^F]FDG PET/CT is generally performed in all the other cases. However, when [^18^F]FDG PET/CT findings are inconclusive or in high-risk patients, we need confirmation of the extent of disease before surgery and we follow a tandem approach consisting of [^18^F]FDG PET/CT plus WBC imaging.

The specific diagnostic flowchart, developed in our institution and currently used for the clinical management of suspected Bentall procedure, is reported in Figure 7. Based on the results of this study, we identified two different subsets: one for very early/early infections and one for all the other infections.

## Discussion

This study evaluated a cohort of patients with a clinical suspicion of infection after the Bentall procedure imaged by either WBC SPECT/CT and/or [^18^F]FDG PET/CT. These results, as for other conditions within the domain of infections, confirmed the high specificity (and PPV) of WBC SPECT/CT and the high sensitivity (and NPV) of [^18^F]FDG PET/CT also in the Bentall procedure setting. Although the relative low probability of infection in this clinical setting (<5%) (32), its management is challenging being burdened by a high mortality. It might: (i) affect to the aortic valve root, (ii) be limited to the vascular aortic graft, or (iii) involve both the aortic valve root and the vascular aortic graft and be extended to the surrounding mediastinal structures including soft tissue and sternum. Evidence about the decisive role of functional imaging in the management of cardiovascular infections has grown over the latest years (1, 33–35). However, as mentioned above, the lack of guidelines to manage patients with infections of the aortic valve root-vascular prosthesis tighter with the peculiarity of this clinical setting makes crucial dedicated criteria to interpret functional imaging. Therefore, on the basis of our extensive experience in multimodality imaging of cardiovascular infections, we proposed to use functional imaging—routinely included in the diagnostic algorithm of infectious endocarditis and prosthetic graft infections—also in patients with a suspected infection after the Bentall procedure. The performance of WBC and [^18^F]FDG PET/CT in this clinical setting was similar to that seen in IE and peripheral VPI (27, 28). PPV of [^18^F]FDG PET/CT was, as expected, relatively low. This result confirmed the clinical need of developing standardized skilled imaging interpretation [^18^F]FDG PET/CT criteria for each specific setting in the attempt to increase the procedure specificity, since it is well-known that [^18^F]FDG is unspecific.

Moreover, as recommended for other cardiovascular infections (31), we evaluated and compared the performance of different criteria [location, pattern, and [^18^F]FDG uptake] to interpret images. No specific pattern of uptake was identified for the diagnosis of infection at the aortic valve component (i.e., IE) similarly to that observed in IE and CIED infections, where both the diffuse and focal pattern uptake associated with a SUVmax > 5 and a valve/lung ratio > 8 were equally able to diagnose IE (36). On the contrary, a focal pattern of uptake with SUVmax > 3 combined with the CT findings was able to improve the specificity of PET/CT at the TG component of the prosthesis with the higher likelihood (specificity 73%).

The evaluation of the pattern of [^18^F]FDG uptake in VPI is crucial to increase the specificity of PET/CT. Keidar et al. (37) demonstrated that non-infected vascular prosthesis presented diffuse [^18^F]FDG uptake in a very high percentage of cases (92%), especially in case of Dacron grafts.

The intensity of [^18^F]FDG uptake in synthetic grafts did not change over time. Since the value of semiquantitative parameters in PET/CT imaging in cardiovascular infection is still a matter of debate in this study, we make the exercise of adding to the visual analysis of the calculation to the most common used semiquantitative assessment in literature. Such findings confirmed that the application of SUV metrics and other semiquantitative parameters, although interesting, are far from being validated in inflammation and infection and they should be used with caution in clinical practice. Indeed, the extrapolation of these cutoff value to other cardiovascular disease states is difficult and the variation in these values related to differences in the scanner and reconstruction methods used should be always considered.

In our series of patients, the specificity of PET/CT increased when considering only patients with non-very early/early infection (<1 month from surgery). A total of 3 out of 22 patients with very early infection had negative WBC SPECT/CT and positive [^18^F]FDG PET/CT (see an example in Figure 4). This datum, consistent with the results reported by Rouzet et al. (38), supported that WBC is not affected by time from surgery and reinforced the evidence that WBC SPECT/CT should be preferred early after surgery.

Appropriate preparation of patients for [^18^F]FDG PET/CT (17, 31, 39–43)—resulting in adequate glucose myocardial uptake suppression—is crucial to interpret images and avoid misdiagnosis, especially to identify the aortic valve root involvement. Prolonged fasting (≥18 h) may improve detection of lesion (44). Blood glucose levels may have a non-linear effect on myocardial uptake and caution should be taken in diabetic patients, since antidiabetic drugs stimulate myocardial glucose uptake. We prepared patients with a low/no carbohydrates fat-enriched diet for 12–24 and 12–18 h fast.

As expected, the performance of both the Duke and the ESC criteria for diagnosis infection after the Bentall procedure was disappointing. The key point is that echocardiography and CT, specifically looking at the cardiac valves and prothesis, respectively, should be sequentially performed in all the cases of infection after the Bentall procedure and in case of their negativity, but high risk, functional imaging could be an efficient option to reduce missed site(s) of infection. However, regardless of the anatomical suspicion of disease in case of the suspected Bentall procedure infectious complication, functional imaging should be not interpreted as in case of IE or vascular prosthesis infection, since the counterpart might be misdiagnosed. Therefore, the application of a comprehensive assessment, as the one proposed in this study, is remarkable—as for IE and other cardiovascular infections (27, 45)—to not underdiagnose infection burden in both the primary and metastatic sites.

We found in up to 34% of patients infection rather than in the prosthetic valve root graft, identifying a cohort of “extended” infection. Accordingly, in all these patients, treatment management was modified and eradication of all the sites of concomitant infections was initiated. Similarly, when surgical indication was present and the clinical condition considered stable surgery was delayed for at least 2 weeks in the attempt to reduce reinfection by specific IV antimicrobial treatment and properly continued after surgery as for specific indication. A further impact of ^99m^Tc-HMPAO-WBC and [^18^F]FDG PET/CT results on management of patients has been documented in 32% of the cases mainly consisting from an intention to treat as best medical treatment to a surgery treatment. However, when a more extent infection in high-risk patients with major surgical contraindication was found, a conservative approach consisting of chronic antimicrobial treatment was supported.

## Limitations

This study has some limitations. Firstly, timing between suspicion of infection and functional imaging was not standardized even if limited (2–8 days from signs/symptoms onset). Nonetheless, all the patients were enrolled in a registry and not in a clinical trial. Moreover, preparation, image acquisition, and postprocessing analysis of patients were performed according to the well-stablished protocols. Secondly, only a subset of patients performed both ^99m^Tc-HMPAO-WBC SPECT/CT and [^18^F]FDG PET/CT, possibly underestimating the impact of these imaging modalities in this specific clinical setting. However, our findings were confirmatory in regard of the high specificity (and PPV) of WBC and the high sensitivity (and NPV) of [^18^F]FDG PET/CT. Furthermore, multimodal imaging—assessing the whole body—informed about possible unexpected foci of infection, even if some inherent pitfalls of each technique should be considered. Lastly, the clinical setting preventing the possibility of proving the efficiency of proposed criteria through a case–control analysis.

## Conclusion

Our findings supported the use of ^99m^Tc-HMPAO-WBC SPECT/CT and [^18^F]FDG PET/CT in patients with high clinical suspicion infection after the Bentall procedure early in the course of the disease onset to confirm the diagnosis and provide a comprehensive assessment of disease burden through the proposed criteria, eventually including multiorgan involvement. By this approach, it is possible to stratify the infection burden and identifying patients with distant sites of infections, contributing to add a unique comprehensive perspective in the multidisciplinary discussion of the subsequent treatment of patients. However, in the subgroup of patients with very early onset of infection after surgery, ^99m^Tc-HMPAO-WBC should be preferred or should complement [^18^F]FDG PET/CT to confirm the findings when conditions of patients allow this tandem approach.

## Data Availability Statement

Raw data are available on specific request to the corresponding author.

## Ethics Statement

The studies involving human participants were reviewed and approved by Comitato Etico Area Vasta Nord Ovest. The patients/participants provided their written informed consent to participate in this study.

## Author Contributions

PE and EL conceptualized the study. PE, EL, and RB designed the study. PE, EL, RZ, and RB screened patients. RB, AC, ML, FM, and MF enrolled, treated, and followed-up patients. FB, RB, and RZ collected the clinical data. PE and EL performed image analysis. PE performed data analysis. EL, MS, PE, RB, AC, ML, FM, and MF critically interpreted results. MS, PE, and FB drafted the manuscript. All authors critically revised the manuscript and approved the submitted version of the manuscript.

## Conflict of Interest

The authors declare that the research was conducted in the absence of any commercial or financial relationships that could be construed as a potential conflict of interest.

## Publisher's Note

All claims expressed in this article are solely those of the authors and do not necessarily represent those of their affiliated organizations, or those of the publisher, the editors and the reviewers. Any product that may be evaluated in this article, or claim that may be made by its manufacturer, is not guaranteed or endorsed by the publisher.
