# Supplementary material for: Role of Multimodal Imaging in Patients With Suspected Infections After the Bentall Procedure
Source: Front Cardiovasc Med. 2021 Dec 1;8:745556. doi: 10.3389/fcvm.2021.745556 (PMC8671629; doi:10.3389/fcvm.2021.745556)
Supplement: Supplementary file 1 [file Data_Sheet_1.PDF]

frontiers

# *Supplementary Material*

## Multimodal imaging in cardiovascular infections: the case of the infectious complications after Bentall procedure.

**M. Sollini[1,2], F Bartoli[3], R. Boni[4], R. Zanca[3], A. Colli[5], M. Levantino[5], F. Menichetti[6], M Ferrari[7], R. Berchiolli[7], E. Lazzeri[3] and P. A. Erba[3,7]**

(1) Department of Biomedical Sciences, Humanitas University, Via Rita Levi Montalcini 4, 20090 Pieve Emanuele, Italy

(2) IRCCS Humanitas Research Hospital, Rozzano (Milan), Italy

(3) Regional Center of Nuclear Medicine, Department of Translational Research and New Technology in Medicine, University of Pisa, and Azienda Ospedaliero Universitaria Pisana, Pisa, Italy

(4) UOC Medicina Nucleare, ASST Papa Giovanni XXIII, Bergamo

(5) Division of Cardiovascular Surgery Department of Surgical, Medical and Molecular Pathology and Critical Care University of Pisa and Azienda Ospedaliero Universitaria Pisana, Pisa, Italy Pisa, Italy

(6) Infectious Diseases Unit, Department of Clinical and Experimental Medicine University of Pisa and Azienda Ospedaliera Universitaria Pisana

(7) Vascular surgery, Department of Translational Research and Advanced Technology in Medicine, University of Pisa, and Azienda Ospedaliero Universitaria Pisana, Pisa, Italy

(8) Department of Nuclear Medicine and Molecular Imaging, Medical Imaging Centre, University Medical Center Groningen, Groningen, The Netherlands

*** Correspondence:**

Paola Anna Erba, MD, PhD

Associated Professor in Nuclear Medicine

Department of Translational research and New Technology in Medicine.

Via Savi 10, 56126 Pisa, Italy

Tel: +39-050-992115

Fax: +39-050-992124

E-mail: p.erba@unipi.it

University of Pisa

Supplementary Table 1: Comparative assessment of the Duke criteria ESC criteria and the New Bentall 2020 criteria in the 76 patients

| | **ESC 2015 Definite (n=23)** | **High AVTG Index (n=56)** | **ESC 2015 Possible (n =18)** | **ESC 2015 Rejected (n=35)** | **Very Low AVTG Index (n=20)** |
|---|---|---|---|---|---|
| **Duke Definite (n=11)** | 11 | 11 | - | - | - |
| **Duke Possible (n=35)** | 9 | 25 | 17 | 8 | 2 |
| **Duke Rejected (n=30)** | 3 | 20 | 1 | 27 | 18 |
